# American Medical Association, Section on Stomatology, Atlantic City, June 7 to 10, 1904

**Published:** 1904-10

**Authors:** 


### Reports of Society Meetings.

### AMERICAN MEDICAL ASSOCIATION, SECTION ON STOMATOLOGY, ATLANTIC CITY, JUNE 7 TO 10, 1904.

(Continued from page 629.)

### DISCUSSION ON THE PAPERS OF DRS. EAMES, BALDWIN, CHITTENDEN, AND MARSHALL, ON DENTAL EDUCATION.

*Dr. N. S. Hoff*, Ann Arbor, Mich.—I do not believe it will do any good at this time to discuss the possibility or the probability of a four years' course of instruction in our dental schools, because the Faculties Association will do what it pleases, and, naturally, to this we shall have to submit. The matter of arranging the course of study in our own school has been my work for several years. Our institution is not very unlike that of Harvard. We have a medical faculty with high ideals, and our work in the line of scientific medical branches is done in the medical school, and our students are compelled to do that work on the same basis as the medical students. I can readily appreciate the situation in which Harvard finds itself. Our school was early forced to go into the four years' course, not because we wanted to get ahead of anybody else, but because our medical school had taken such advanced ground on the scientific subjects that we were compelled to have our students instructed in those departments by a proper amount of time. What shall be the position of our school in view of the position of the Faculties Association, I am not prepared to say, but I do not see how it will be possible for us to do our work short of a four-year basis; and I think that if things go on as they have in the medical department, we shall be driven to a five years' course. My impression is that by our plan of working we are not developing our course symmetrically. The scheme that I have been turning over in my mind is that in the first year our students should take up the technic work, and as fast as possible they should be advanced through the practical courses, at the same time carrying sufficient of the scientific branches to keep up the mental discipline which they have already acquired by their high-school training. We admit students from high schools, but they are all examined, and we know what their teaching has been. We require two years of Latin, if the student has had only one language. We prefer that he has one modern language, preferably German. The majority of our students from the high school come at the age of nineteen, which it seems to me is not an excessively mature age for a man to take up technical training. I believe such men will develop their technical training more rapidly if taken up at that age than if it is delayed until later in the course; and, if we can advance them more rapidly at that period, why not do it, and at the same time allow them to carry, not full work in the scientific departments, but only sufficient amount to keep them in mental drill; for instance, the study of chemistry and anatomy may be properly carried along, because they do not need to see the relations of chemistry at the beginning. They will be able to complete the subject of chemistry later on in the course. I was talking with the deans of two medical colleges in the West in which they have dental departments, and who complained of the difficulty in getting dental work done. They asked me how we did at Ann Arbor. I said, "We don't do it; it is a physical impossibility to get a medical man who has never had any dental training to interest a dental practitioner in the subject from a theoretical stand-point. He must know something practical about it that will call the student's attention to the work. I have found this true in therapeutics; the students could answer me according to the text-books, but they had no idea of the relations to practical work. I do not know how they will make that application of their work, unless we first teach them to be practical dentists. Then when they come to these physiologic principles they will see the relation to their own work. I remember that it took me a long while to apply my one hundred per cent. chemistry in my examination record to my dental chemistry. I think the profession recognizes the fact that if there is a standard it must not be dogmatic, for there are different classes, and I do not think it is necessary for the schools in Class A to take into consideration the schools in Class B. The standard is sentimental, and it is the greatest power for the uplift of our profession. It is this unwritten standard, or whatever it may be called, that I am looking for. I think it will be thoroughly discouraging, if the course of study is put back to three years, or if a lower standard is made by the

Association of Faculties. It will take years for us to get back to where we now are. I do not believe the subject has ever been studied from a proper stand-point. I have given you the stand-point from my own view.

*Dr. Alice M. Steeves,* Boston.—My idea is that the dental student does not work from the idea of benefiting the entire body; he does not see the relationship very often. I think we should impress on him that he is working for the benefit of the whole and not for one individual.

*Dr. Bogue,* New York City.—The chairman and Dr. Hoff have assisted in crystallizing my incoherent views. My boy, who had promised me he would study dentistry, felt that he wanted to study medicine. I said to him, "I claim the fulfilment of that promise. When you have done that, all you can earn you may devote to the study of medicine." He worked so hard that after graduation in dentistry he had already passed his first year in medicine. He afterwards graduated in medicine, and in that obtained what I had been desiring for years,—that he should know when he began the study of medicine what he wanted to get out of that study. He could not do that until he had graduated as a dentist. He received his operative practice at the University of Pennsylvania, where individual ability I found could be recognized, and got this practice at an earlier age than he could have gotten it at either of the other schools. It has been demonstrated to us how the musician beginning at an early age becomes technically so perfect that his fingers accomplish the thought of his mind almost automatically. Let that musician begin later on in life and it can never be done. From that stand-point I draw a parallel with the dentist's work, and I believe that unless a dentist begins his technical work comparatively early in life he will never succeed in his profession. Now, what is his profession, gentlemen? Suppose this room-full of practitioners were called in an hour from now to attend a case of broken lower jaw, how many of us are perfectly qualified to take that case in hand? How many of us would treat a case of cleft palate? I am sure I would not dare undertake Brown's operation. Right there is another cause that should call this body into relationship with the great medical body. I allude to these things, because it seems to me they have a bearing on the curriculum yet to be decided on. There are things in dental education which have been left behind which we should have with us, and one is private tutelage before the student ever undertakes to enter college. I have spent some time in the class-rooms in Harvard, and in those of other colleges, and have noted the difference in what I have seen of the students. I do not say that the classes graduated at Harvard would be any better able to do the work that a dental surgeon ought to feel himself called on to do than those from other schools; but I do know all too sadly that the dental graduate as a whole is not the man he ought to be. He is not sufficiently qualified by any manner of means to take charge of the oral cavity and to keep it in health.

*Dr. M. L. Rhein*, New York City.—These papers and this discussion and all the literature of our profession on this subject are most conclusive evidence of the correct assumption of our chairman in his paper on the crying need of symmetry in general education. My own impression in this matter is that the cause of the trouble is not in the professional education. The source is much deeper. It is in the primary methods, or pretences at methods of education to which the world has been accustomed; the education of youth from childhood up is in a very great progress of evolution at the present time, and has been progressing materially. It is impossible for us to arrive at any of the ideals that Professor Truman or Professor Hoff would like to see at the present time, and the main trouble is at the primary education. I differ entirely with the theory of Dr. Hoff, and which Dr. Bogue advanced, of taking up the general medical education later. That will not cure the trouble. The trouble can be reached if the education of youth is properly conducted. The greatest progress in this direction is the induction of manual training in the primary education. Manual training brought to its proper level is the true solution of this problem. If a child in its primary education has received the proper manual training, so that he becomes deft enough with his fingers as a child to do a piece of wood-carving or the work of the other departments, it will be as impossible for him to lose this deftness as it will be impossible for him to lose his skill on the ice, so easily acquired in childhood and so difficult in later life. Another thought which is uppermost in my mind in this direction is that it is impossible to make dentists. We can aid in the education of different classes of dentists, from very good ones, to certain inferior ones; and yet, out of certain material, we must recognize that it is impossible to produce any sort of dentists. The fact that so much of that material is brought into the profession with the degree of D.D.S. attached to the name is not to the credit of the institution that such men should have passed three years there when it is self-evident that they are unfit to ever become successful practitioners. In view of this fact, last year, I was led to say that I was strongly in favor of getting rid of all the poor colleges at any cost; that I thought the ultimate interest of the profession would be enhanced if any means were used to annihilate what is known as the commercial institute in dentistry. It is in these institutions that such material is allowed to go through. I have no doubt that some of it gets through the better grade of institution, but in no such proportion. I have stood aghast at the position Harvard has taken in this matter. It is to me one of the most inconceivable things for an institution of that character to do. I sympathize with everything that Dr. Briggs has stated to us as being the position of Harvard, but it is no excuse for Harvard's action. I agree with Dr. Briggs that it may be a matter of discussion whether it is better to advance the preliminary education, or whether it is better to advance the course, but that discussion should have been entered into long before the actual meeting. There were two years of the discussion before the meeting of the Association of Faculties, when Harvard had an opportunity to consider that subject in a way that she failed to do, or to lead any one to suppose that she would take the position that would be utilized by the commercial institutions for the degradation of dentistry. No one fails to realize that Harvard is not allied with them, but they take advantage of the position which Harvard has taken. As an earnest advocate for the highest advancement of dental education, I do not believe that it would suffer one iota if at the meeting at Washington a large number of the colleges would secede from the four years' course. I believe it would result in the annihilation of the commercial colleges, because the examining boards are in favor of the four years' course. Another point of interest was that referred to by Dr. Hoff,—the difficulty in securing instruction to the dental student from the medical men. That is another point where education requires a great deal of remedying in the future; that is, lack of proper education, not only of the medical teacher, but of the medical student, because the medical student must ultimately become the teacher. If he knows nothing about the mouth he is not qualified to teach the groundwork of medical practice to future dentists or stomatologists.

Another point, and one which has been eloquently dilated on by Dr. Marshall: We know that we are not willing to take a position lower in the scale of the medical men, and yet we are placed in that position, because we have failed to keep up to the trend of evolution in medical education. Is there any reason why our education should be inferior to that of the medical institution? None in the least. Dr. Briggs is right there. That is where Harvard is right, and we are wrong. It is not only necessary for us to stand for a four years' course, but we must not present them with a calling that places dental practitioners on a more inferior basis than the medical men, if we would attract to our specialty the best of the youth of this country. We do that the moment we lower the standard of our entrance examinations in our institutions. I would like to emphasize this on the departments of the universities who are interested in the real uplift of the educational standard. It matters little how many students they may lose in a matter of this kind. It matters much whether they elevate the character of the material that is attracted to us. One of the curses of the general education to-day is that in the lower branches the teaching is uniform. The minds of the children are trained alike, and yet they are all totally different.

It is impossible to satisfy the desire of our friend Dr. Bogue in telling how we shall make the model dental education. I have simply tried to bring up a few of the defects; but I want to say that if the manual dexterity is acquired in childhood, the basic principles which should precede specialization can not fail to be properly acquired. I want to introduce a resolution as part of the discussion of this subject, as follows:

*Resolved,* The Section on Stomatology of the American Medical Association, in session at Atlantic City, sends its greetings to the National Association of Dental Faculties. We congratulate the Association on the completion of the first year of the advanced four years' course. We sincerely trust that having the honor and standing of the profession in your hands, no action will be taken that will tend to lower the advanced stand that has been taken.

I would like to have this Section send to them this expression and our hope that they will not falter in the position taken.

On motion, the resolution was adopted.

*Dr. William Lederer,* New York City.—Dr. Rhein said correctly, "Dentists are born." To my mind dentistry is both a science and an art. The man who is a scientist alone or the one who is a craftsman and an artist alone is not a dentist. Whether a course in a school is three or four years, that will not make him a better or a worse man. To have ideal conditions and to further dental education two factors are necessary, just as two factors are necessary to produce a work of art,—the artist and the material which he turns into a work of art. If the most ideal conditions prevail in the institutions, and the material which is entered is not capable of properly imbibing the teachings, the result will not be good dentists. Theory can be taught, but mechanical ability can not be taught, and, therefore, I should think the essential training would be a combination of the practice and the teaching. It is stated that there is only one school in this country whose degree enables a man to practise in Germany, and that is Ann Arbor. I am sure we have other schools in this country which turn out as able men as Ann Arbor. How they place the standard I do not know. Ann Arbor is a State institution. If some movement were started to create State institutions which can not be commercial, perhaps that would solve the problem, and we would have proper material and proper artists to do a work of art.

*Dr. Edward C. Kirk.*—I agree with Dr. Rhein that the great defect in the mind, in the career, in the qualification of the dental student for acquiring his education is the fault of his training in the kindergarten. I do not believe it is because the method is uniform, but because the method is faulty. You cannot make a mind elective that has not the power of election. That is what parents are for. Education is to be of use. We study arithmetic, and we go on farther with the relations of numbers until we get into higher mathematics. Only those whose calling demands the use of mathematics employ them. There is another use for mathematics,—the mental discipline. That is to get into the mind of that human being an appreciation of the fact that two and two make four—not three and seven-eighths or four and a quarter. It is to develop a respect for precision as an element of character. The great fault in the dental student is that he is not precise and does not reason logically and accurately. I had a talk with my colleague, Dr. Truman, on the bad use of English and the misspelling of these men. I kept in my examination markings a list of terms misspelled by American-born and educated students of our high schools. Dr. Truman tells me that it is a psychic state developed by the examination stress. He is very tender-hearted with his delinquents. I believe the training of the dentist should be begun in the kindergarten. There is room for us to suggest improvement in the methods of preliminary education. I agree also with Dr. Rhein that it is unsafe in the making of a dentist to postpone his manual training, and such manual training should be specific; it must be related to his calling as a dentist. I do not agree that a man should take a medical training as preparatory to dental education. I am of the opinion that we must superadd whatever he needs in the medical training. I also believe it is a mistake for a student to have his preparatory training in a dental office. Such training should be post-graduate. Under the present arrangement of the college curriculum we have the methods for training, which is a better plan than the old apprenticeship system. I think that any one who has looked at this thing conscientiously and who knows anything about the subject will agree that it is impossible to produce a dentist worthy of the name in less than four years under present conditions. I believe the whole reason why there is a desire on the part of the faculties (I except Harvard; I understand her position, and it has nothing to do with my remark; she has adopted a different plan of arriving at the same end) to revert to the three year's course is purely a commercial one. I know that there would have been no opposition to the four years' course had there not been a drop in the Freshmen classes of from fifty to seventy-five per cent. in many instances. That recalls to my mind one point referred to by the essayist,—that as a purely commercial proposition it pays to maintain the highest possible standard. There are enough decent, honest people in the world to back up such an effort, and on the principle that honesty is the best policy, even in the absence of any other moral consideration, it would pay. The main reason is, of course, that the four years' course makes better dentists.

*Dr. George V. I. Brown.*—I believe I am the real culprit. Mine happened to be the resolution passed in our Faculty Association for the four years' course. I believed in it very thoroughly then, and I believe in it even more now. I believe Harvard's going out last year was a mistake, and I hope that before very long the Association and Harvard will meet on common ground. The criticism of the kindergarten system refers to matters which we can not change. We have to deal with students who have been educated under a system, good or bad, that has been in existence, and it is well for us to help in rearranging the future. I believe we must and will have a four years' course. No matter whether one man who is more highly educated than another can learn more in six months or a year than the other, we must base our standard on the average. There is no question that the more a man trains his fingers in dental work the better, and no one will question that he can do more of that in four years than three. I believe that if we are doing our duty we will have a personal interest in the men under our care, and I believe we can do more to uplift them ethically in four years than in three. I have had to face the proposition of trying to make a dental student do in one year the same work that medical men were doing and do forty hours a week beside, and I found he could not do it. I am hopeful about this Faculty Association meeting. When the final issue has come on any question there have always been enough good men to carry what is right. So far as Harvard's position is concerned, the University of Iowa, Ann Arbor, and I daresay Pennsylvania, will have more than a full year more of instruction than they, but we are willing to suffer that disadvantage—if it is a disadvantage—for the sake of training much better men.

*Dr. Eugene S. Talbot,* Chicago.—I am glad that Harvard has taken the position she has. I am in favor of the four years' course, and I am sure that there is a great deal of good going to come out of this peculiar condition in which the profession stands. It is only by this friction that we arrive at results. I am going to discuss this subject on a little different plan from what most have spoken. They have been talking along the mechanics of dentistry. The question comes up at the present time, Is there not another side than the mechanics of dentistry? There was not when the first dental school was established. But it seems to me we know a little more along the line of stomatology than we knew sixty-four years ago, and the question arises, Are the dental colleges keeping pace with our present knowledge? We have been talking of the practice of dentistry as it is to-day. What will become of the practice of dentistry sixty-four years from now, if it progresses as it has in the last sixty-four years? Are we going to remain as mechanics and manipulators? It seems to me that there are two conditions at the present time that are uppermost in the minds of the dental profession,—decay of teeth and interstitial gingivitis. We have been studying decay of teeth as a local condition, and we have advanced far enough in the last few years to know that disease of the human body has a great deal to do with decay of teeth. We know that it has much to do with the saliva of the mouth, that it produces a change in the saliva. We know that pregnancy has much to do with decay of the teeth, also typhoid fever and pneumonia. Yet at the present time, although we have been teaching sixty-four years, we have not gotten down to the first principles of decay of the teeth and interstitial gingivitis. There is not a single dental college teaching the principles of the nervous system. What have we to say in regard to interstitial gingivitis? What do we know about intestinal fermentation, the great cause of gingivitis? I want to say to the teachers here that we have yet to learn the first principles of teaching these diseases, and we have got to introduce them in our schools. Until we have educated students we must have educated men in order that we can teach the students the diseases of the human body. We come to the point that some school, and I hope it will be Harvard, will take the lead and will require, first an academic degree; second, two years in pathology in our medical schools, and third, manipulation; and as soon as some university will teach men to become teachers to fill our dental colleges with men who are capable of teaching, we shall have better dental students.

*Dr. G. V. I. Brown.*—I agree with Dr. Talbot that we should have more of the teaching to which he refers. We have men here who have been teaching for years, and Dr. Talbot has said we do not teach about nervous diseases. I should like to know their statements on this question.

*Dr. Talbot.*—There are no chairs on the pathology of the nervous system.

*Dr. Kirk.*—We teach the nervous system, but I understood the secretary to lay down rather dogmatically that the perverted nervous system had as much to do with interstitial gingivitis, if not more than any other factor, except the local conditions. I think that is rather a broad statement, and there comes to the mind the proposition of which came first, the egg or the hen. There is such a thing as faulty metabolism, and that probably has something to do with the perverted nervous system. We have not taught that faulty action of the nervous system is the fundamental error, but we teach fundamentally that faulty metabolism is back of the whole disturbance.

*Dr. E. C. Briggs,* Boston.—I do not want you to think that Harvard does not want four years, or that she is standing out against it. That never has been the point. I think the time is coming when Harvard will demand four years. The time is coming when she will demand other things. While we felt that we could not take every step at once, we felt that the step which we did take was the next step to take. I can not deny the accusation that Dr. Rhein made that this thing ought to have been threshed out before the motion was carried to make four terms the course. It seemed to me that Dr. Truman's remarks, stating that there were some students whom he did not like to see go out as dentists, are an argument for my point. You can not help getting men in who are not fitted, and the men who go out are safer if they have had a good preliminary education.

*Dr. A. E. Baldwin,* Chicago.—The question is not one of degrees; what we want is the education. Dr. Talbot is correct in his statement that we should pay more attention to the definite causes of the conditions and not content ourselves with the manipulative end. We have too many men in the profession whose minds go no farther back than the work and the correction they can give it. I do not think there is a man practising dentistry to-day or being prepared for practice who is not the better for the broadest kind of an education. In the medical school I saw as much evidence of manipulative skill as in the dental college. At the same time, I do not mean to belittle the manipulative part of the education taught in our dental colleges. We do not occupy a progressive field when we talk about going back to the pupilage system. That can not go farther than the men. If the institutions are what we suppose them to be they are a combination of the teaching of the best men, and to go from them to the office of some fossilized person—of which, perhaps, I would be one—would be very foolish. I have seen men of broad education who were failures in their chosen vocation, but I do not lay it to the broad education. There are some men who have a wonderful lack of education and who are yet wonderful successes. Education is not a method of cramming things in, but it is the drawing out of what is within. We will have some failures even with four, or a ten years' course, but we shall have more intellectual work done.

*Dr. M. L. Rhein.*—I am sure that all the gentlemen who spoke of the manipulative point in educational acquirements had no intention of objecting to the phase presented by Drs. Baldwin and Talbot. We realize that all the scientific attainment possible is valueless without the manipulative ability in our specialty. The two must go hand in hand. The point is that it is impossible to instil that manipulative ability if it is lacking in the personality of the individual, and one of the defects in our institutions is the absence of a method by which applicants may be received for a probationary period, and if it is found at the end of this time that they are out of their sphere, they may be rejected.

*Dr. George F. Eames,* Boston.—Dr. Baldwin has stated that education is a drawing out of what is within. My idea would be to ascertain the bent of a child while in the kindergarten and in his later education, taking a broad view to determine his probable choice of professions. Then when he has arrived at the age when he knows what he wants there will be some data of value. It is not a degree; it is not a number of years, but it is the qualification of the individual to be in our specialty, and an American citizen, and when he has developed equally and symmetrically in all of these lines, he has attained the object, and it is for us to decide the time spent in preliminary education, and how much within the college walls. I believe a man who has it within him to be a success, must escape the bonds and rules of an institution by the time he is twenty-five years of age. We have not a lease on life. If we increase the length of the course, we do not have the life in proportion.

